# Expression analysis of the endogenous *Zscan4* locus and its coding proteins in mouse ES cells and preimplantation embryos

**DOI:** 10.1007/s11626-016-0097-y

**Published:** 2016-10-03

**Authors:** Kei-ichiro Ishiguro, Yuhki Nakatake, Nana Chikazawa-Nohtomi, Hiromi Kimura, Tomohiko Akiyama, Mayumi Oda, Shigeru B.H. Ko, Minoru S.H. Ko

**Affiliations:** 1 Department of Systems Medicine, Keio University School of Medicine, 35 Shinanomachi, Shinjuku, Tokyo, 160-8582 Japan; 2 Institute of Molecular Embryology and Genetics, Kumamoto University, 2-2-1 Honjo, Chuo-ku, Kumamoto, 860-0811 Japan

**Keywords:** Zscan4, ES cell, Preimplantation embryo, Two-cell stage, Knock-in

## Abstract

Mouse Zinc finger and SCAN domain containing 4 (Zscan4) is encoded in multiple copies of *Zscan4* genes, which are expressed in late two-cell stage preimplantation embryos and in 1–5% of the embryonic stem (ES) cell population at a given time. Due to the highly identical nucleotide sequences of multiple copies of *Zscan4* paralogs and pseudogenes in the mouse *Zscan4* genomic cluster, previous analyses have been done using exogenous transgenes under the regulation of *Zscan4c* promoter. In this manuscript, we generated knock-in mouse ES cell lines and mouse lines, in which the expression of endogenous *Zscan4c*, one of the *Zscan4* genes, can be specifically monitored with a green fluorescent protein variant, Emerald. Interestingly, we found that only ∼30% of Zscan4-immunopositive ES cells were Emerald positive, suggesting that even when the Zscan4 locus is active, not all *Zscan4* genes are expressed synchronously. We also carried out mass spectrometry of protein complexes associated with endogenous Zscan4 proteins. Taken together, our genetic engineering at an endogenous *Zscan4c* gene provides the first clue for the expression and function of each gene copy of *Zscan4* locus in a physiological context.

## Introduction

Zinc finger and SCAN domain containing 4 (*Zscan4*) genes were originally identified as genes that are specifically expressed during zygotic genome activation (ZGA) in the late two-cell stage of mouse preimplantation embryos (Falco et al. 2007). The mouse genome encodes nine members of the *Zscan4* paralogs (*Zscan4a*, *Zscan4b*, *Zscan4c*, *Zscan4d*, *Zscan4e*, and *Zscan4f* and three pseudogenes *Zscan4-ps1*, *Zscan4-ps2*, and *Zscan4-ps3*). In the human genome, there is only one copy of *Zscan4* gene (Falco et al. 2007). Among the mouse *Zscan4* genes, *Zscan4c*, *Zscan4d*, and *Zscan4f* encode a full-length 506-aa protein, whereas *Zscan4a*, *Zscan4b*, and *Zscan4e* encode truncated proteins (360 amino acids (aa), 195 aa, and 195 aa, respectively) (Falco et al. 2007). In two-cell stage embryos, the knockdown of *Zscan4* by small interfering RNA (siRNA) leads to a delay of progression from the two-cell to four-cell stage and, consequently, implantation failure (Falco et al. 2007)*.* In mouse embryonic stem (ES) cells, the expression of *Zscan4* is transient and reversible with infrequent transcriptional activation in only 1–5% of the cell population at a given time point (Falco et al. 2007) (Zalzman et al. 2010). A burst of *Zscan4* transcription (Z4 events) is accompanied by biological events including transient expression of other ZGA-specific genes (Amano et al. 2013; Akiyama et al. 2015) rapid derepression and rerepression of heterochromatin regions (Akiyama et al. 2015), rapid telomere extension (Zalzman et al. 2010), and blockage of global translation (Hung et al. 2013). Additionally, Zscan4 has also been shown to enhance the efficiency of generating mouse-induced pluripotent stem (iPS) cells and their quality (Hirata et al. 2012; Jiang et al. 2013). These data suggest that Zscan4 plays diverse biological roles during Z4 events of ES cells and in two-cell stage preimplantation embryos.

In the previous studies, Z4 events were mostly identified in ES cells with a reporter transgene, in which the fluorescent reporter expression is under an artificial *Zscan4c* promoter region (Zalzman et al. 2010; Akiyama et al. 2015)*.* However, a potential issue that has yet to be clarified is whether the minimum 3.6-kb genomic fragment of the putative *Zscan4c* promoter region mirrors the bona fide expression pattern of the endogenous *Zscan4* locus due to random integration in the genome, copy number effect, and any missing *cis*-regulatory element. In addition, due to limited material available for biochemical examination, it has been elusive which member of the endogenous Zscan4 proteins act for Z4 events in ES cells and for preimplantation embryo development, although previous RNA-sequencing analyses suggest that all the members of *Zscan4* messenger RNA (mRNA) are expressed (Akiyama et al. 2015), albeit *Zscan4c* is expressed predominantly in ES cells, and *Zscan4d* is expressed predominantly in two-cell stage embryos (Falco et al. 2007). Furthermore, attempts to genetically modify any given *Zscan4* locus by conventional gene targeting have been technically hampered due to the highly identical nucleotide sequences of multiple copies of *Zscan4* genes and pseudogenes in the *Zscan4* genomic cluster. This has been an obstacle for genetic study of the *Zscan4* genes.

In this manuscript, we successfully generated ES cell lines and mouse lines with an *Emerald-GFP (Emerald)* knock-in allele at the *Zscan4c* locus by using CRISPR/hSpCas9 (Cong et al. 2013) specifically targeting the *Zscan4c* genomic locus. The established knock-in ES cell lines and mouse lines allowed us to dissect the bona fide expression pattern of *Zscan4c* and actions of the locus to external stimuli in the context of the endogenous locus in ES cells and two-cell stage embryos*.* Moreover, combined with mass spectrometry, the knock-in ES cells facilitated analysis of the endogenous Zscan4 protein and its associated factors. Thus, genetically engineered knock-in ES cells at a given *Zscan4* locus will shed light on further approaches—not only to study the roles of individual *Zscan4* members but also to analyze the knockout of *Zscan4* gene clusters in a physiological context.

## Materials and Methods

### **Embryonic stem cell culture**

TA1 mouse ES cells (F1 hybrid of C57BL/6J × 129S6/SvEvTac) and the derivative cells were used for all experiments unless otherwise specified (Amano et al. 2013). During the establishment of recombinant ES clones, the cells were initially cultured in 2i+LIF condition (Millipore, Bedford, MA) on the MMC-treated MEF feeder cells. For experiments, ES cell lines were maintained on gelatin-coated feeder-free plates in complete ES medium (Zalzman et al. 2010). For experiments using retinoic acid (RA), all-trans-RA was added at a final concentration of 1 μM in the complete ES medium. Two independent Silencer select siRNA against Zscan4 (Thermo, Kanagawa Japan: s233511, s233512) and negative control siRNA (Thermo: AM4611) were used to prepare Zscan4-depleted and control mESC extracts.

### **Generation of*Zscan4c-Emerald*knock-in embryonic stem cells and genotyping**

The targeting vector was designed to replace Exon 2 and the following intron of the *Zscan4c* genomic locus with *Emerald*-polyA followed by *Neo* cassette. The targeting arms of 3.56- and 2.66-kb fragments, 5′ and 3′, to the *Zscan4c* gene, respectively, were generated by PCR from C57BL/6 genomic DNA and directionally cloned in pKOII plasmid, flanking a pGK-Neo-polyA, a *loxP* site, and a DT-A cassette. The homologous recombinant cells were isolated using TA1 ES cells (F1 hybrid of C57BL/6J × 129S6/SvEvTac) after transfection of the targeting vector together with CRISPR/Cas9 pX330-U6-Chimeric_BB-CBh-hSpCas9 vectors (Addgene, Cambridge, MA) encoding specific guide RNAs 5′-GCCUGUGAUCUGUGGAAGUG-3′ and 5′-CCCACACUUCCACAGAUCAC-3′, which direct the intron between Exon 2 and Exon 3 of the *Zscan4c* genomic locus. The G418-resistant ES clones were screened for homologous recombination in the *Zscan4c* locus by PCR using primers, Zscan4c (Z4c)-7206F 5′-AGAATGATATGTGTAGCCATATTAC-3′ and Z4c-Em10892R 5′-AACAGCTCCTCGCCCTTGCTCACCATG-3′ for 5′-arm (3687 bp) and Z4c-Neo-13534F 5′-GCTATCAGGACATAGCGTTGGCTAC-3′ and Z4c-mut16545R 5′-AACAAGCCAAAATTAAAAGGGTTAG-3′ for 3′-arm (3422 bp). Since the *Zscan4* genomic cluster consists of multiple copies of *Zscan4* genes and pseudogenes with high nucleotide sequence identity, correct targeting at *Zscan4c* genomic locus was verified by direct sequencing of the PCR products of 5′-arm and 3′-arm regions of the *Zscan4c* locus for the presence of the specific single-nucleotide differences among *Zscan4* loci and fluorescence in situ hybridization (FISH) using a probe encompassing *Emerald* and *Neo* genes.

### **Generation of*Zscan4c-Emerald*knock-in mice**

The chimeric mice were generated by blastocyst injection (host ICR) of recombinant *Zscan4c-Emerald-Neo* + knock-in ES cells. The male chimeric mice were crossed with C57BL/6J females to generate the F1 offspring. To confirm germline transmission of the *Zscan4c-Emerald-Neo*+ knock-in allele, genotyping was performed by PCR using primers Z4c-Neo-13534F 5′-GCTATCAGGACATAGCGTTGGCTAC-3′ and Z4c-13786Rmut 5′-CTTGTCCTTGCATTGAAACATGGAC-3′ for 3′-arm (597 bp) and Z4c-10503Fwt 5′-GATACAGGACTTTCTGGTTATTGAG-3′ and Z4c-Em10892R 5′-AACAGCTCCTCGCCCTTGCTCACCATG-3′ for 5′-arm (363 bp). The knock-in mice were congenic with the C57BL/6J background. Whenever possible, each knock-in animal was compared to littermates or age-matched non-littermates from the same colony, unless otherwise described. A transgenic mouse expressing Flippase, D2-Tg(CAG-FLP)18lmeg (from Dr. Masatake Araki, Kumamoto University, CARD), was crossed with a *Zscan4c-Emerald-Neo* (*Neo* allele) knock-in mouse to delete pGK-Neo-polyA cassette franked by Frt sequences, producing *Zscan4c-Emerald Neo*Δ knock-in allele (*Neo*Δ allele). For genotyping of *Zscan4c-Emerald* NeoΔ knock-in allele, PCR was performed using primers Z4c-11852F 5′-GGCGGCAGGCCCTGCCATAGCCATG-3′ and Z4c-13786Rmut 5′-CTTGTCCTTGCATTGAAACATGGAC-3′, producing a 261-bp band. Due to the highly identical nucleotide sequences of *Zscan4* multigene loci, wild-type *Zscan4c* locus cannot be distinguished from other *Zscan4* loci by PCR genotyping. Thus, to identify homozygous *Zscan4c-Emerald* knock-in siblings, a heterozygous *Zscan4c-Emerald*-*Neo* knock-in mouse (*Neo*/+) was crossed with a heterozygous *Zscan4c-Emerald*-*Neo*Δ knock-in mouse (*Neo*Δ/+). A homozygous *Zscan4c-Emerald* knock-in mouse (*Neo*/*Neo*Δ) was identified by genomic PCR for the presence of both *Neo*Δ and *Neo* allele. Animal experiments were approved by the Institutional Animal Care and Use Committee (approval no. 12702–0).

### **Embryo collection and culture**

Females were injected with 5 U of PMSG (Merck Millipore, Tokyo Japan) and 48 h later with 5 U of hCG (Calbiochem). Embryos were flushed out of the mouse oviducts in pxotassium simplex optimization medium (KSOM) medium 38 h after hCG injection. Embryos were cultured in KSOM medium.

### **Fluorescence in situ hybridization**

ES cells grown on a cover glass were fixed in 4% paraformaldehyde for 5 min; washed with PBS; and subjected to sequential dehydration through 70, 80, 90, and 100% ethanol, denatured in 50% formamide and 2× saline-sodium citrate (SSC) at 72°C for 10 min. Hybridization was conducted with a FITC-labeled probe that detects *Emerald GFP* and *Neo* derived from the knock-in vector, in buffer containing 50% formamide, 2× SSC, 20% dextran sulfate, and 0.1 μg/μl of mouse Cot-1 DNA at 37°C for 16 h. The cover glasses were washed sequentially at room temperature in 2× SSC for 1 min, 0.4× SSC/0.3% Tween 20 solution for 2 min, and 2× SSC at room temperature for 1 min. The FITC-labeled probe was generated by nick translation (Hokkaido chromosome science) using equimolar mixture of two templates, *Emerald GFP* and *Neo*. DNA fragments of *Emerald GFP* and *Neo* were generated by PCR, using primers Z4c-Emerald (Em)-FISH-F 5′-atggtgagcaagggcgaggagctgttc-3′ and Z4c-Em-FISH-R2 5′-gctatggcagggcctgccgccccgacg-3′ for *Emerald GFP* (1006 bp) and Neo-1F 5′-ATGGGATCGGCCATTGAACAAGATG-3′ and Neo-801R 5′-GAAGAACTCGTCAAGAAGGCGATAG-3′ for *Neo* (801 bp). Nuclei were counter stained with 4′,6-diamidino-2-phenylindole (DAPI).

### **In situ hybridization followed by immunostaining**

Antisense *Zscan4c* RNA was synthesized by in vitro transcription with T3 RNA polymerase, using DIG-UTP (Roche, Tokyo Japan) and a mouse *Zscan4c* complementary DNA (cDNA) template cloned in pBluescript KS+. DIG-labeled antisense *Zscan4c* RNA probe was fragmented by alkaline (3: 2 mixture of 0.2 M Na_2_CO_3_/0.2 M NaHCO_3_) into 300–500 base at 60°C for 15 min. The ES cells grown on a cover glass were fixed in 4% paraformaldehyde for 5 min, washed with PBS and 0.1% Triton X-100/PBS, and subjected to acetylation by acetic anhydride in 0.1 M triethanolamine (pH 8.0). Hybridization was conducted in 50% formamide, 2× SSC, 1 mg/ml tRNA, 1 mg/ml salmon sperm DNA, 1 mg/ml bovine serum albumin (BSA), and 10% dextran sulfate with DIG-labeled antisense *Zscan4c* RNA probe at 37°C for 12 h. After washing with NTE buffer (0.5 M NaCl, 10 mM Tris-Cl [pH 8.0], 1 mM EDTA), the unhybridized RNA probe was removed by 20 μg/ml RNase A in NTE buffer. After washing with 0.1× SSC and blocking with 1% digoxigenin (DIG) detection buffer (Roche), immunodetection was done by mouse anti-DIG and rabbit anti-mZscan4 followed by fluorescent second antibodies.

### **Production of antibodies**

Polyclonal antibodies against mouse Zscan4 (aa 1–506) were produced by inserting cDNA fragment in-frame with pET19b (Novagen, Gibbstown, NJ) in *Escherichia coli* strain BL21-CodonPlus # (DE3). His-tagged recombinant proteins were solubilized in a denaturing buffer (6 M HCl-guanidine, 20 mM Tris-HCl [pH 7.5]) from the inclusion body and purified by Ni-NTA (Qiagen, Hilden, Germany) under denaturing conditions. After dialyzing against PBS, the purified protein was used to immunize rabbits. The antibodies were affinity-purified from the immunized crude serum with immobilized antigen on CNBr-activated Sepharose (GE Healthcare, Piscataway, NJ).

### **Preparation of chromatin extracts**

To prepare chromatin extracts, Emerald-positive mouse ES cells were enriched by fluorescence-activated cell sorting (FACS) sorting and suspended in the extraction buffer (20 mM Tris-HCl [pH 7.5], 100 mM KCl, 0.4 mM EDTA, 0.1% Triton X-100, 10% glycerol, 1 mM β-mercaptoethanol) supplemented with Complete Protease Inhibitor (Roche). After Dounce homogenization, the soluble cytoplasmic/chromatin-unbound fraction was separated after centrifugation at 100,000*g* for 30 min. The insoluble pellet was washed two times with buffer (10 mM Tris-HCl [pH 7.5], 1 mM CaCl_2_, 1.5 mM MgCl_2_, 10% glycerol) and digested with micrococcus nuclease (0.008 units/ml) at 4°C for 60 min. The solubilized fraction was removed after centrifugation for 15 min at 4°C. The chromatin fraction was extracted from the insoluble pellet by high-salt extraction buffer (20 mM HEPES-KOH [pH 7.0], 400 mM KCl, 5 mM MgCl_2_, 0.1% Tween 20, 10% glycerol, 1 mM β-mercaptoethanol) supplemented with Complete Protease Inhibitor. The solubilized chromatin fraction was collected after centrifugation at 100,000*g* for 30 min at 4°C.

### **Immunoprecipitation**

For immunoprecipitation of endogenous mouse Zscan4 from chromatin fraction, 5 μg of affinity-purified rabbit anti-mZscan4 and control rabbit IgG antibodies was cross-linked to 50 μl of protein A-Dynabeads by DMP (Sigma). The antibody-cross-linked beads were added to the solubilized chromatin extracts, and the extracts were incubated for 2 h at 4°C with rotation. The beads were washed with high-salt extraction buffer. The bead-bound proteins were eluted with 40 μl of elution buffer (100 mM glycine-HCl [pH 2.5], 150 mM NaCl) and then neutralized with 4 μl of 1 M Tris-HCl [pH 8.0].

### **Mass spectrometry**

For mass spectrometry analysis of the endogenous mouse Zscan4 and its associated proteins, the immunoprecipitates were run on the 10% NuPAGE gel (Thermo Fisher Scientific, Waltham, MA) and separated 2 cm from the well. The gel was excised and washed with 25 mM ammonium bicarbonate followed by acetonitrile. The proteins were reduced with 10 mM DTT at 60°C for 30 min followed by alkylation with 50 mM iodoacetamide and subjected to in-gel digestion by trypsin gold (Promega, Madison, WI) at 37°C for 4 h. The gel digests were analyzed by nano-LC-mass spectrometry (MS)/MS with a Waters NanoAcquity HPLC system interfaced to a Thermo Fisher Orbitrap Velos Pro (MS Bioworks, Ann Arbor, MI). Data were searched using Mascot with the following parameters: database: Swissprot Mouse (forward and reverse appended with common contaminants); fixed modification: carbamidomethyl (C); variable modifications: oxidation (M), acetyl (Protein N-term), Pyro-Glu (N-term Q), and deamidation (NQ); mass values: monoisotopic; peptide mass tolerance 10 ppm; fragment mass tolerance 0.6 Da; and max missed cleavages 2. Mascot DAT files were parsed into the Scaffold software for validation, filtering, and creating a non-redundant list per sample. Data were filtered at 1% protein and peptide false discovery rate and requiring at least two unique peptides per protein. Those proteins were determined as most significantly elevated in the Zscan4 immunoprecipitation (IP) sample versus the control IgG sample based on the following criteria: Protein had at least five spectral counts (SpC) in the Zscan4 IP sample. Protein was not detected in the control IgG sample or detected with a fourfold or more increase based on dividing the SpC values.

### Fluorescence-activated cell sorting

The fluorescent intensity analysis and sorting of Emerald of Z4c-Em ES cells were performed using a BD FACSAria II. The cells were sorted according to the fluorescent intensity of Emerald and collected into mouse ES cell culture medium at 4°C.

### **Immunofluorescence staining**

ES cells were grown on a cover glass and fixed in 4% PFA for 5 min at room temperature and permeabilized in 0.1% Triton X-100 in PBS for 10 min. The cells were blocked for 10 min in PBS and 3% BSA and incubated at room temperature with the primary antibodies in a blocking solution. After three washes in PBS, the cells were incubated for 1 h at room temperature with Alexa-dye-conjugated secondary antibodies (1:1000; Invitrogen) in a blocking solution. DNA was counterstained with Vectashield mounting medium containing DAPI (Vector Laboratories).

### **Imaging**

Immunostaining images were captured with DeltaVision and processed with DeltaVision SoftWorx software (GE Healthcare). All the images shown were Z-stacked. Green fluorescent protein (GFP) fluorescence and bright field images were captured with an OLYMPUS IX73 fluorescence microscope and processed with the CellSens standard program.

### **Antibodies**

The following antibodies were used: mouse anti-GFP (Santa Cruz, Santa Cruz, CA: sc-9996), rabbit anti-GFP (Abcam, Cambridge, UK: ab6556), mouse anti-DIG (Abcam: ab6212), and rabbit anti-actin (CST no. 4970).

## Results

### **Infrequent activation of the endogenous mouse*Zscan4c*promoter in the knock-in embryonic stem cells**

Our previous examination of the fluorescent reporter expression from the *Zscan4c* promoter*-Emerald* transgene showed that the *Zscan4c* promoter was transiently activated in only 1–5% of MC1 mouse ES cells (pZscan4-Emerald cell) at a given time point (Zalzman et al. 2010; Akiyama et al. 2015). However, a potential concern was yet to be clarified: whether or not the *Zscan4c* promoter*-Emerald* transgene reflects the bona fide expression pattern of *Zscan4* locus due to random integration in the genome, copy number effect, and missing *cis*-regulatory elements in the transgene construct. Thus, we generated an ES cell line in which the Emerald fluorescent reporter is placed at the endogenous *Zscan4c* locus to examine its expression pattern in the context of the endogenous promoter and regulatory element in ES cells and mouse embryos*.*

**Figure 1.**
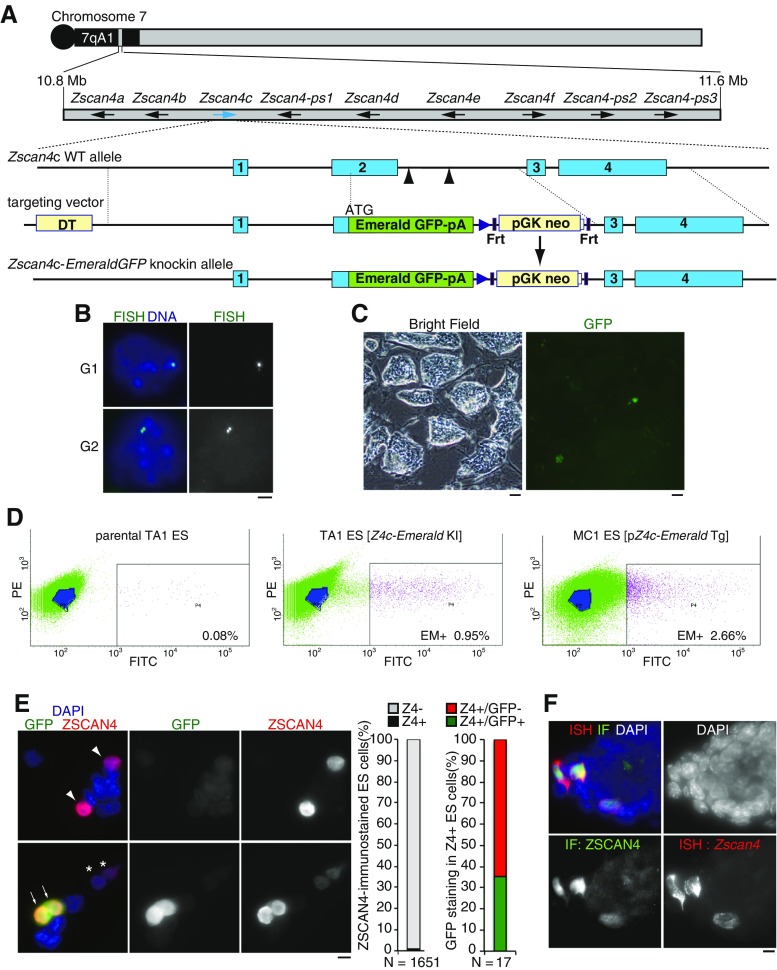
Targeted knock-in of *Emerald GFP* reporter in *Zscan4* gene cluster reveals endogenous activity of *Zscan4c* locus in ES cells. (*A*) Schematic illustrations of the 7qA1 region of mouse chromosome 7, where multiple *Zscan4* genes (*Zscan4a∼f)* and pseudogenes (*Zscan4-ps1∼3*) cluster (*arrows*: direction of transcription from each *Zscan4* gene or pseudogene). The wild-type *Zscan4c* allele, *Zscan4c-Emerald* knock-in (*Z4c-Emerald*-KI) allele, and targeting vector are shown. Targeted replacement of Exon 2 and the following intron of the *Zscan4c* locus with *Emerald GFP* results in Z4c-Emerald-KI allele, in which *Emerald GFP* is under an endogenous *Zscan4c* promoter. *Blue boxes* represent exons. *Black boxes* represent Frt sites. *Blue triangles* represent lox P sites. *Arrowheads* indicate the targeting sites of guide RNAs for an hSpCas9-mediated DNA double-strand break. (*B*) FISH using an FITC-labeled probe that specifically detects the portion of *Emerald GFP*-*Neo* in the knock-in allele showed a *single dot* or a *pair of single dots* (two juxtaposed signals derived from sister chromatids) before or after the S phase, respectively, indicating that one allele of *Zscan4c* was replaced by the knock-in vector in the Z4c-Emerald-KI ES clone. *Scale bar*, 1 μm. (*C*) Emerald+ cells were infrequently observed in the Z4c-Emerald-KI ES colonies. *Left*: bright-field image; *right*: GFP fluorescent image. *Scale bar*s, 50 μm. (*D*) Fluorescence-activated cell sorting (FACS) analyses of Emerald+ (EM+) ES population in the parental TA1 ES cells (*left*, negative control), the Z4c-Emerald-KI ES cell clone no. 27 (*middle*), and MC1 pZ4c-Emerald-Tg ES cells (*right*) in the conventional (FBS+ LIF) ES culture condition. Note that the overall scatter plot in MC1 pZ4c-Emerald-Tg ES cells shifts toward the right, indicating that the overall Emerald intensity is higher compared to that in Z4c-Emerald-KI ES cells. (*E*) *Zscan4c-Emerald GFP* knock-in ES cells were immunostained as indicated using rabbit anti-Zscan4. *Arrows*: GFP+/Zscan4+ cell; *arrowheads*: GFP−/Zscan4+ cell; *asterisks* GFP weakly+/Zscan4 weakly+ cell. *Scale bar*, 10 μm. Zscan4/GFP-immunostained ES cells were quantified, showing that 1.03% of the total ES cells were Zscan4+ (*left graph*) and 35.3% of Zscan4-immunopositive cells were GFP+/Zscan4+ (*right graph*). None of the GFP+/Zscan4-cells was observed. (*F*) ES cells were subjected to in situ hybridization of bulk *Zscan4* mRNA (ISH, *red*) and immunostaining by rabbit anti-Zscan4 antibody (IF, *green*), demonstrating that anti-Zscan4 immunopositivity correlates with the expression of bulk *Zscan4* mRNA. Note that bulk *Zscan4* mRNA is detected over cytosol, while the Zscan4 protein is immunostained in nuclei. *Scale bar*, 10 μm.

Our previous attempts to generate genetically engineered alleles at any given *Zscan4* locus by conventional gene targeting methods failed repeatedly, most likely due to the highly identical nucleotide sequences of multiple copies of *Zscan4* paralogs and pseudogenes in the mouse *Zscan4* genomic cluster (Fig. 1 *A*). However, by using CRISPR/hSpCas9 combined with chimeric guide RNAs specifically targeting the *Zscan4c* genomic locus, we successfully established an ES cell line with *Emerald* knock-in allele at the *Zscan4c* locus (Z4c-Emerald-knock-in (KI) ES cell), where Exon 2 and the following intron were replaced by *Emerald*-polyA (Fig. 1 *A*). FISH showed that one *Zscan4c* allele was knocked in, whereas the other *Zscan4c* allele was intact in the Z4c-Emerald-KI ES cell (Fig. 1 *B*), indicating that the knock-in ES cell line is heterozygous in terms of *Zscan4c* allele.

Emerald-positive (Emerald+) cells were infrequently observed in the Z4c-Emerald-KI ES colonies (Fig. 1 *C*), indicating that the endogenous *Zscan4c* allele was activated in a limited population. FACS analysis indicated that approximately 0.95% of the Z4c-Emerald-KI ES cells were Emerald+ in the feeder-free conventional mouse ES culture condition, whereas parental cells rarely showed Emerald fluorescence (Fig. 1 *D*), essentially recapitulating our previous observation that a small subpopulation was Emerald+ in MC1 Z4c-Emerald-transgene (Tg) ES cells (Zalzman et al. 2010). However, we noted that the frequency and fluorescence intensity of Emerald+ cells in the Z4c-Emerald-KI ES cells were less than those observed in MC1 Z4c-Emerald-Tg ES cells (∼2.6%) in the same culture condition (Fig. 1 *D*). The apparent difference between Z4c-Emerald-KI ES cells and Z4c-Emerald-Tg ES cells could be accounted for partly by the genetic background of mouse ES cell lines: Z4c-Emerald-KI ES cells (F1 hybrid of C57BL/6 and 129) and MC1 Z4c-Emerald-Tg ES cells (129 line), as we have shown previously (Sharova et al. 2007; Amano et al. 2013).

Crucially, coimmunostaining of Z4c-Emerald-KI ES cells by anti-Emerald and anti-Zscan4 antibodies revealed that only a fraction (∼35%) of Zscan4-immunopositive cells showed Emerald immunopositivity (Fig. 1 *E*), thus indicating that counting Emerald+ cells underestimates the actual total number of Zscan4-positive (Zscan4+) cells. Although we have not assessed precisely the protein turnover of the Emerald GFP in the Z4c-Emerald-KI ES cells, Emerald+ embryonic stem cells (ESCs) always show Zscan4 immunopositivity in the Z4c-Emerald-KI ES cells, indicating that Emerald can monitor the activation of at least endogenous Zscan4c locus in real time. Thus, although anti-Emerald immunofluorescence intensity was correlated with that of anti-Zscan4, it should be noted that Emerald was not expressed in all the Zscan4+ ES cells. This observation seems to be paradoxical, given the evidence that our previous RNA-sequencing analyses showed that all of the *Zscan4* paralog genes were expressed in FACS-sorted Emerald+ ES cells (Akiyama et al. 2015). Nevertheless, since our newly generated anti-Zscan4 polyclonal antibody cross-reacts with all of the mouse Zscan4 protein members (at least Zscan4c, Zscan4d, and Zscan4f—see also Fig. 4) and Zscan4 immunopositivity correlates with bulk expression of *Zscan4* paralog mRNA (Fig. 1 *F*), the aforementioned observation rather suggests that the *Zscan4c-Emerald* knock-in locus is not always activated in a given Zscan4-immunopositive cell. Thus, it is possible that not all of the *Zscan4* loci together but a subset of them in the *Zscan4* multigene cluster shows transient transcriptional firing at a given time window of a Z4 event in mouse ES cells.

### **Endogenous mouse*Zscan4*genes redundantly function during mouse two-cell stage preimplantation development**

**Figure 2.**
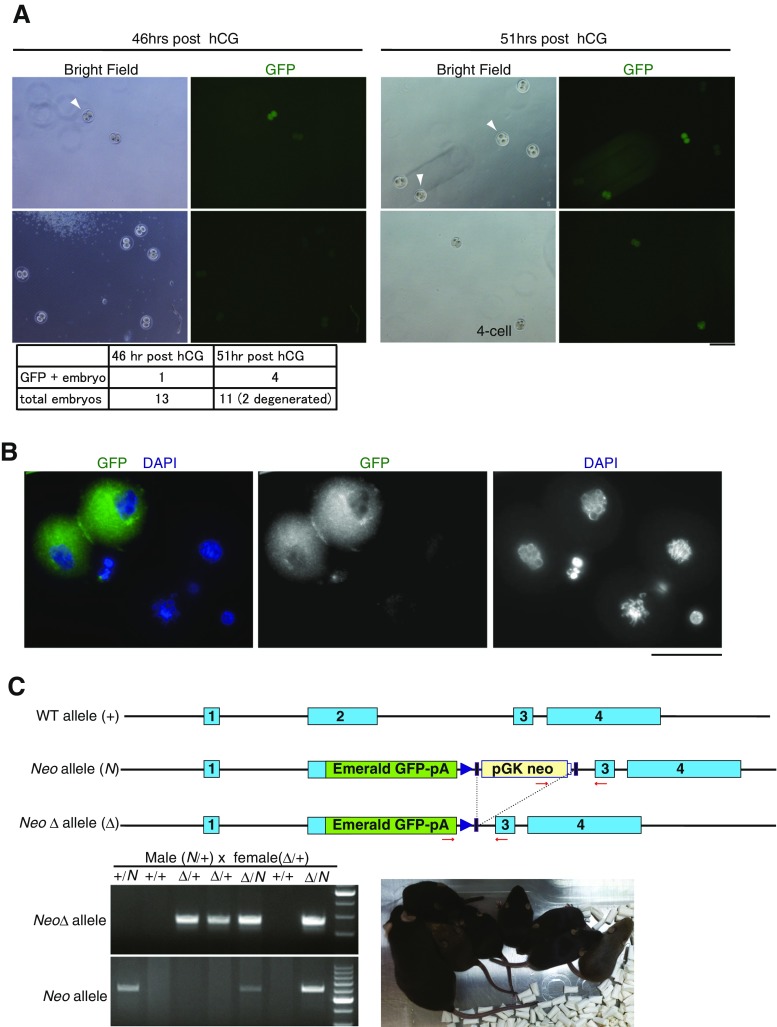
The *Zscan4c-Emerald* GFP reporter knock-in mouse revealed endogenous activities of *Zscan4* loci in the mouse preimplantation embryo. (*A*) GFP fluorescent images of two-cell stage embryos collected from a heterozygous *Z4c-Emerald*-KI female mouse crossed with a wild-type male at 46 h (*upper left*) and 51 h (*upper right*) post hCG. The *white arrowhead* indicates Emerald+ two-cell embryo. The number of Emerald+ two-cell embryos is summarized in the table (*lower*). *Scale bar*, 200 μm. (*B*) The late two-cell stage embryos at post 51 h hCG were immunostained with anti-GFP. *Z4c-Emerald* KI two-cell embryo showed GFP staining, while polar bodies or wild-type two-cell embryos did not. *Scale bar*, 50 μm. (C) Schematic illustrations of the wild-type *Zscan4c* allele (+), *Zscan4c-Emerald GFP-Neo* knock-in allele (*Neo*), and *Zscan4c-Emerald GFP-Neo*Δ knock-in allele (*Neo*Δ) are shown (*upper*). The *Neo*Δ allele knock-in mouse line was generated by crossing a *Neo* knock-in mouse with a transgenic mouse expressing Flippase to delete pGK-Neo-polyA franked by Frt sequences. *Arrows*: PCR primes. Due to the highly identical nucleotide nature of *Zscan4* multigene loci, wild-type *Zscan4c* locus cannot be distinguished from other *Zscan4* loci by PCR genotyping. To facilitate identification of homozygous *Zscan4c-Emerald* knock-in siblings, *Neo*/+ mouse was crossed with *Neo*Δ/+, producing homozygous *Neo*/*Neo*Δ, which was identified by PCR (*lower left*). Homozygous *Neo*/*Neo*Δ siblings, in which functional *Zscan4c* allele is disrupted, are viable (*lower right*).

Originally, *Zscan4* was identified as a gene specifically expressed in the mouse two-cell stage embryo (Falco et al. 2007). Previous RT-PCR suggested that *Zscan4d* was predominantly expressed among the *Zscan4* genes in the two-cell stage embryo, but *Zscan4c* was also expressed (Falco et al. 2007). To confirm this notion, we generated a knock-in mouse line with a *Z4c-Emerald*-KI allele from the recombinant ES clone to examine the expression pattern of the endogenous *Zscan4c* locus in preimplantation embryos. The two-cell stage embryos were collected from heterozygous *Z4c-Emerald*-KI mice crossed with wild type, half of which should be theoretically heterozygous for *Z4c-Emerald*-KI. This allowed for a parallel comparison of fluorescence between wild-type and heterozygous *Z4c-Emerald*-KI embryos in the exact same condition. Although *Z4c-Emerald*-KI two-cell stage embryos showed less intense fluorescence compared to Emerald+ ES cells, probably due to diffusion over the larger size of the cell, 36% of two-cell embryos showed Emerald fluorescence at 51 h post hCG administration (Fig. 2 *A*), which is roughly the expected ratio for heterozygous *Z4c-Emerald*-KI embryos. The number of Emerald+ two-cell embryos increased at 51 h post hCG compared to that at 46 h, suggesting that *Z4c-Emerald*-KI allele is fully activated in the late rather than the early two-cell stage, consistent with our previous observation by in situ hybridization (Falco et al. 2007). This observation was further confirmed by immunostaining of late two-cell embryos with the anti-Emerald antibody, showing that the *Z4c-Emerald*-KI late two-cell embryo, but not the associated polar bodies, is immunopositive for Emerald (Fig. 2 *B*). Thus, the endogenous mouse *Zscan4c* allele is activated during the embryonic two-cell stage. These results emphasize that the *Z4c-Emerald*-KI allele recapitulates the stage-specific expression pattern of the endogenous *Zscan4c* gene in the mouse preimplantation embryo.

Importantly, we noted that mice with a homozygous *Zscan4c-Emerald*-KI allele, which is supposed to be a knockout of functional *Zscan4c* genes, were still viable without any obvious defect (Fig. 2 *C*). Moreover, there is no overt phenotypic difference in the reproduction and development between heterozygous and homozygous knock-in mice (Fig. 2 *C*), suggesting that loss of functional *Zscan4c* genes was compensated by other *Zscan4* loci—most likely *Zscan4d*—during embryonic development. Thus, it is suggested that not only *Zscan4c* but also other *Zscan4* genes act redundantly for preimplantation embryonic development.

### **The endogenous mouse*Zscan4c*promoter is activated by retinoic acid in the knock-in embryonic stem cells**

It has been demonstrated that expression of *Zscan4* is positively regulated by telomere shortening (Nakai-Futatsugi and Niwa 2016), DNA damage, and PI3 kinase-mediated external signals (Storm et al. 2014) and is negatively regulated by Tet (Lu et al. 2014), Rif1 (Dan et al. 2014), and Nr01b (Fujii et al. 2015). However, the molecular details of how an infrequent burst of endogenous *Zscan4* expression is triggered remain largely unknown.

**Figure 3.**
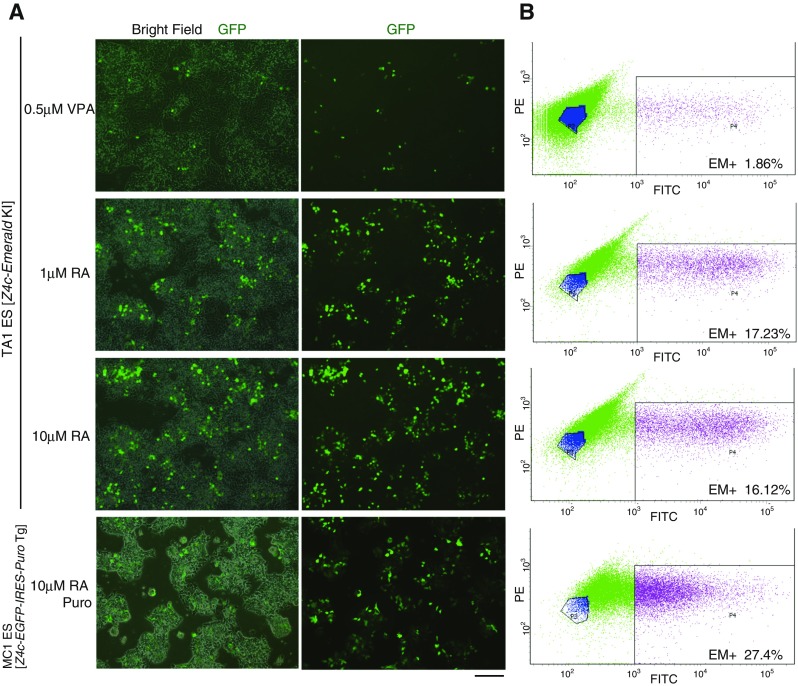
The *Zscan4c*-*Emerald GFP* reporter knock-in locus is activated by retinoic acid in ES cells. (*A*) GFP fluorescence images of the *Z4c-Emerald*-KI ES cells in the presence of VPA (0.5 μM) or RA (1 μM, 10 μM) and of the MC1 ES cells with *Z4c-GFP-IRES-Puro* transgene in the presence of RA (10 μM) and puromycin. *Scale bar*, 200 μm. (*B*) FACS analyses of the GFP+ population in the *Z4c-Emerald*-KI ES cells and MC1 *Z4c-GFP-IRES-Puro* Tg ES cells as indicated in (*A*). Note that the overall scatter plot in *Z4c-GFP-IRES-Puro* Tg ES cells, which acquire puromycin resistance only when GFP is expressed, shifts toward the right, indicating that the overall GFP intensity of the survived cells selected against puromycin is higher compared to that in *Z4c-Emerald*-KI ES cells.

Our previous study demonstrated that the burst of transcription at Z4 events coincides with transient histone hyperacetylation (Akiyama et al. 2015). Thus, we examined whether an HDAC inhibitor triggers activation of the *Z4c-Emerald* knock-in locus. FACS analysis indicated that Emerald+ ES cells were slightly increased (Fig. 3 *A*, *B* compared with Fig. 1 *D*) in the presence of the HDAC inhibitor, valproic acid (VPA), suggesting that histone acetylation slightly promotes activation of the *Z4c-Emerald*-KI locus.

Given that retinoic acid (RA) activates Zscan4 expression (Sharova et al. 2007) (Sharova et al. 2016), we examined the expression of *Emerald* from *Z4c-Emerald*-KI locus after RA treatment. Remarkably, FACS analysis indicated that approximately 16–17% of the *Z4c-Emerald*-KI ES cells were Emerald+ in the presence of RA for 48 h (Fig. 3 *A*, *B*), suggesting that the endogenous *Zscan4c* locus is directly or indirectly activated in response to RA. To exclude a possibility of auto-fluorescence derived from dead or dying cells, we assessed the RA effect on MC1 ES cells with the *Zscan4c* promoter*-EGFP-IRES-puro* transgene (MC1 *Z4c-EGFP-IRES-Puro* Tg ES cell), which acquire puromycin resistance upon EGFP expression (Fig. 3 *A*, *B*). Because the Zscan4c-activated cells survived against puromycin selection and showed EGFP fluorescence with high intensity, we can exclude a possibility that dying or dead cells with auto-fluorescence were detected after RA treatment. Although physiological relevance of RA signaling for activation of the endogenous *Zscan4* locus requires further investigation, the endogenous *Zscan4* locus might have a *cis*-regulatory element that responds to RA or relevant stimuli. Thus, the *Zscan4* locus acts as a sensor to cell-extrinsic and cell-intrinsic stimuli in ES cells.

### **Expression of endogenous Zscan4 proteins and their associated factors in embryonic stem cells**

**Figure 4.**
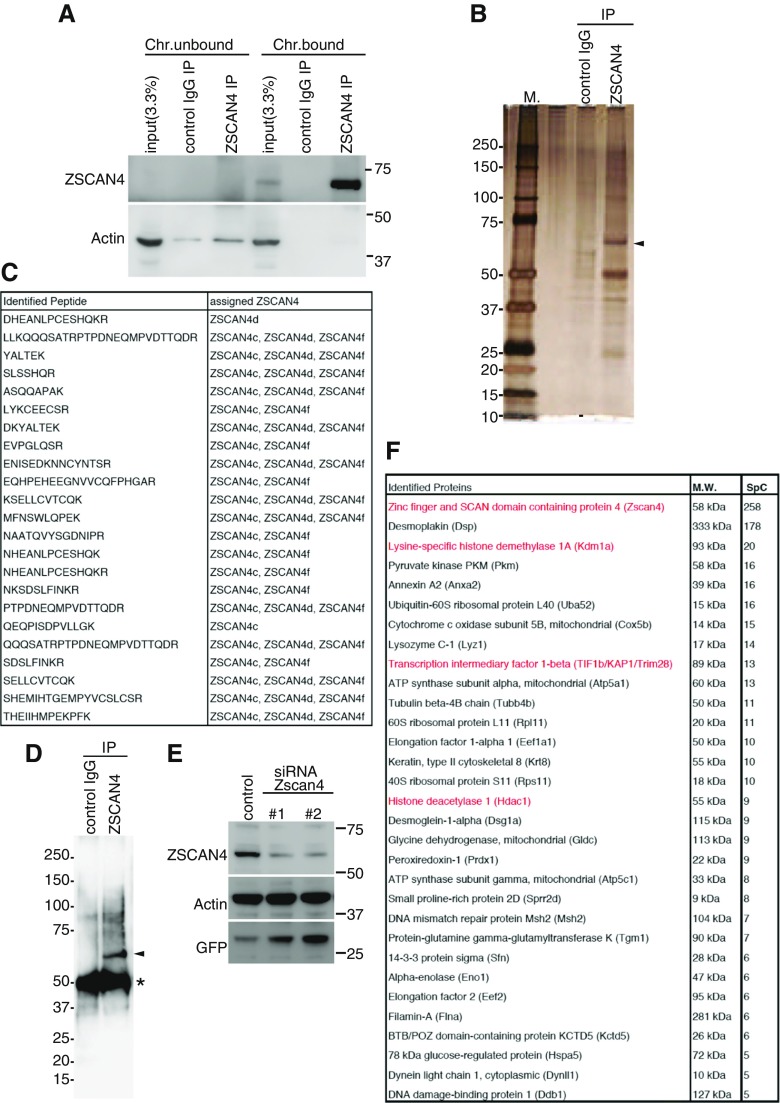
Mass spectrometry analysis revealed the expression of endogenous Zscan4 proteins and their associated factors in Z4c-Emerald knock-in ES cells. (*A*) Emerald+ mouse ES cells after RA treatment for 48 h were separated into chromatin bound and unbound fractions. Immunoprecipitation (IP) was done by control IgG and rabbit anti-Zscan4 antibody from chromatin-bound and chromatin-unbound fractions. Western blotting showed that endogenous Zscan4 protein was exclusively extracted from the chromatin-bound fraction. (*B*) Silver-stained gel showing immunoprecipitates (IP) by control IgG and rabbit anti-Zscan4 antibody from the chromatin fraction of Emerald+ ES cells, enriched by FACS sorting after RA treatment. *M*: molecular weight marker. *Arrowhead*: Zscan4. (*C*) LC-MS/MS analysis of immunoprecipitated endogenous Zscan4 showing unique MS spectrums specifically assigned to peptide sequences derived from Zscan4c, Zscan4d, or Zscan4f. (*D*) Western blotting of immunoprecipitated endogenous Zscan4 probed with rabbit anti-Zscan4 antibody. *Arrowhead*: Zscan4. *Asterisk*: IgG heavy chain. (*E*) Whole-cell extracts of RA-treated Z4c-Emerald KI ES cells after administration of *Zscan4* or negative control siRNA were probed with antibodies as indicated. Note that specificity of the immunoreactive band detected by rabbit anti-Zscan4 antibody was confirmed by siRNA against *Zscan4* genes. (*F*) List of proteins identified in the Zscan4 immunoprecipitates by LC-MS/MS analysis. Spectral counts (SpC) of the identified peptide are indicated. Highlighted in *red* are the Zscan4-associated factors identified in the previous MS analysis of Zscan4c-FLAG immunoprecipitates after overexpression (Akiyama et al. 2015).

Our previous RNA-sequencing analyses showed that all the *Zscan4* genes were activated during the Z4 event (Akiyama et al. 2015). Due to limited available material, however, it remained elusive which members of the endogenous Zscan4 proteins are expressed in ES cells. Moreover, Zscan4 proteins show high amino acid sequence identity, which hampers the technical ability to identify an individual Zscan4 protein member by anti-Zscan4 antibody, which was previously raised against the most C-terminal peptide (14aa) epitope of Zscan4c (Zalzman et al. 2010). Nevertheless, the evidence that RA enhances Emerald reporter expression from the knock-in allele prompted us to collect Emerald+ ES cells by FACS sorting and examine the endogenous Zscan4 protein. For this analysis, we generated new polyclonal antibodies against the entire full length (506 aa) of the Zscan4 protein since the previously raised anti-Zscan4 antibody (Zalzman et al. 2010) may have missed detection of the truncated types of Zscan4a, Zscan4b, and Zscan4e proteins. Our biochemical fractionation followed by immunoprecipitation demonstrated that the endogenous Zscan4 protein was mostly separated into the chromatin-bound fraction extracted from Emerald+ ES cells (Fig. 4 *A*), suggesting that the endogenous Zscan4 protein is tightly associated with chromatin rather than free from it.

Intriguingly, MS analysis of the immunoprecipitated endogenous Zscan4 identified unique MS spectrums, which were specifically assigned to trypsin-digested peptide sequences derived from Zscan4c, Zscan4d, or Zscan4f (Fig. 4 *B*, *C*). Notably, the identified MS spectrums showed more Zscan4c peptides than other Zscan4 peptides (Fig. 4 *C*), suggesting that the Zscan4c protein could be dominantly expressed among the Zscan4 proteins in the Emerald+ ES cell population, in agreement with predominant expression of *Zscan4c* mRNA (Falco et al. 2007). By contrast, this analysis identified none of the peptides assigned to truncated type members (Zscan4a, Zscan4b, or Zscan4e), despite redundant coverage of peptides from Zscan4c, Zscan4d, or Zscan4f. Consistent with this observation, immunoprecipitation followed by Western blotting of Zscan4 showed that only a single band, which corresponds to larger forms (Zscan4c, Zscan4d, or Zscan4f) but not to putative truncated forms (Zscan4a, Zscan4b, or Zscan4e), was detectable (Fig. 4 *D*). Moreover, siRNAs against *Zscan4* genes confirmed that this immunoreactive band is specific to Zscan4 (Fig. 4 *E*). Thus, these results suggest that a subset of different Zscan4 members is indeed expressed at a protein level in Emerald+ ES cells, while expression of the putative truncated form of Zscan4 is less than detectable level.

A previous overexpression study using a doxycycline-inducible transgene of FLAG-Zscan4c showed that Zscan4c is associated with transcriptional repressors and activators (Storm et al. 2014; Akiyama et al. 2015). However, factors associated with endogenous Zscan4 are yet to be analyzed despite physiological relevance. Our MS analysis of the immunoprecipitated endogenous Zscan4 identified potential Zscan4-associated factors (Fig. 4 *F*). Consistent with our previous report using the exogenous FLAG-Zscan4c (Storm et al. 2014; Akiyama et al. 2015), the data indicate that transcriptional repressors KDM1A/LSD1, KAP1/TIF1β, and HDAC1 are associated with endogenous Zscan4, supporting our previous observation that Zscan4 acts for transcriptional repression at heterochromatic regions (Akiyama et al. 2015).

## Discussion

The repetitive nature of the multiple *Zscan4* genes in mouse and their strictly limited expression patterns have hampered genetic analysis of a *Zscan4* locus in physiological conditions. Here, the CRISPR/Cas9 technique, specifically directed at a *Zscan4c* locus, allowed us to scrutinize bona fide expression patterns and mode of action to external stimuli of endogenous *Zscan4c* gene in ES cells and two-cell stage embryos*.* Because we employed conventional CRISPR/Cas9 system in this study, we cannot exclude a possibility of off-target cuts and indels in the established ESC clones. However, it is unlikely that this concern affects our analyses of Zscan4 locus, because (i) different clones of the knock-in ESC essentially recapitulated the same character in terms of Emerald expression and (ii) the established knock-in mouse lines were viable.

Intriguingly, our study uncovered that the *Zscan4c-Emerald* knock-in locus is not always activated in a given Zscan4+ ES cell (Fig. 1 *F*), suggesting that a subset of *Zscan4* loci in the *Zscan4* multigene cluster is expressed at a given time of the Z4 event in mouse ES cells. It was previously shown that expression of *Zscan4* is activated by DNA damage, PI3 kinase-mediated signal (Storm et al. 2014), and RAs (Sharova et al. 2007; Sharova et al. 2016) and suppressed by DNA cytosine demethylation status (Lu et al. 2014), telomeric factor Rif1 (Dan et al. 2014), and nuclear receptor Nr01b (Fujii et al. 2015). Similarly, we demonstrated that endogenous *Zscan4* expression is triggered in response to extrinsic stimuli in ES cells (Fig. 3). Therefore, the implication for those observations is that the *Zscan4* locus is imposed under several layers of regulations that respond to multiple intrinsic and extrinsic stimuli in ES cells and probably during ZGA in two-cell stage embryos. Thus, the molecular details regarding how the burst of endogenous *Zscan4* expression is acquired in ES cells and embryos await further investigation.

Supporting previous overexpression studies (Akiyama et al. 2015), we have demonstrated that the endogenous Zscan4 protein is bound to chromatin in association with KDM1A/LSD1, KAP1/TIF1β, and HDAC1 (Fig. 4), as has been implicated in the rapid derepression and rerepression of heterochromatin regions (Akiyama et al. 2015). It is worth noting that robust phosphorylation of KAP1/TIF1β by ATM is a critical determinant for 53BP1-mediated DNA damage repair in the heterochromatic region, which is otherwise inhibitory to repair (Noon et al. 2010). Given that the Zscan4+ cell is accompanied by arrest or accumulation at the G2 phase of the cell cycle in ES cells (Nakai-Futatsugi and Niwa 2016) (Storm et al. 2014) and late two-cell stage embryos (Fig. 2) (Falco et al. 2007), it is possible that Zscan4 plays pivotal roles not only in transcriptional regulation but also in a checkpoint response to heterochromatic regions for stable genome integrity.

Taken together, our genetically engineered knock-in ES cells provided insight into modes of action at a *Zscan4* locus. Combining with CRISPR/Cas9-mediated targeting at a different *Zscan4* gene, e.g., Zscan4d, in this locus will allow us not only to study the biological roles of individual *Zscan4* members but also to analyze the knockout phenotype of the *Zscan4* gene cluster in ES cells and preimplantation embryos.

## Conclusion

We newly generated knock-in mouse ES cell lines and mouse lines, in which the expression of endogenous *Zscan4c*, one of the *Zscan4* genes, can be specifically monitored with a green fluorescent protein. Our genetic engineering at the endogenous *Zscan4c* gene allowed the first analysis for the expression and function of each gene copy of *Zscan4* locus in a physiological context, providing previously unforeseen phenomena by *Zscan4-*transgene studies. Our data showed that only ∼30% of Zscan4-immunopositive ES cells were Emerald positive, suggesting that even when a given *Zscan4* locus is active, not all endogenous *Zscan4* genes are expressed synchronously. Our MS of protein complexes associated with endogenous Zscan4 proteins showed that Zscan4 acts as a transcriptional repressor in a physiological context.
